# *Babesia Bovis* Ligand-Receptor Interaction: AMA-1 Contains Small Regions Governing Bovine Erythrocyte Binding

**DOI:** 10.3390/ijms22020714

**Published:** 2021-01-13

**Authors:** Laura Cuy-Chaparro, Michel David Bohórquez, Gabriela Arévalo-Pinzón, Jeimmy Johana Castañeda-Ramírez, Carlos Fernando Suárez, Laura Pabón, Diego Ordóñez, Gina Marcela Gallego-López, Carlos Esteban Suárez, Darwin Andrés Moreno-Pérez, Manuel Alfonso Patarroyo

**Affiliations:** 1 Molecular Biology and Immunology Department, Fundación Instituto de Inmunología de Colombia (FIDIC), Carrera 50#26-20, Bogotá DC 111321, Colombia; lauracuy@outlook.com (L.C.-C.); mdbohorqueza@unal.edu.co (M.D.B.); 2 PhD Programme in Biomedical and Biological Sciences, Universidad del Rosario, Carrera 24#63C-69, Bogotá DC 111221, Colombia; 3 Receptor-Ligand Department, Fundación Instituto de Inmunología de Colombia (FIDIC), Carrera 50#26-20, Bogotá DC 111321, Colombia; gabarpi@gmail.com (G.A.-P.); jeimmyjohana93@gmail.com (J.J.C.-R.); 4 Biomathematics Department, Fundación Instituto de Inmunología de Colombia (FIDIC), Carrera 50#26-20, Bogotá DC 111321, Colombia; cfsuarezm@gmail.com; 5 Chemistry Department, Fundación Instituto de Inmunología de Colombia (FIDIC), Carrera 50#26-20, Bogotá DC 111321, Colombia; mlpabona@gmail.com; 6 Animal Science Faculty, Universidad de Ciencias Aplicadas y Ambientales (U.D.C.A.), Calle 222#55-37, Bogotá DC 111166, Colombia; dordonez@udca.edu.co; 7 Department of Medical Microbiology and Immunology, University of Wisconsin—Madison, Madison, WI 53706, USA; gimagalo@gmail.com; 8 Morgridge Institute for Research, Madison, WI 53715, USA; 9 Animal Disease Research Unit, USDA-ARS, 3003 ADBF, WSU, P.O. Box 647030, Pullman, WA 99164-6630, USA; suarez@wsu.edu; 10 Microbiology Department, Faculty of Medicine, Universidad Nacional de Colombia, Carrera 45#26-85, Bogotá DC 111321, Colombia

**Keywords:** *Babesia bovis*, AMA-1, minimum region, adhesion, bovine erythrocyte, synthetic vaccine

## Abstract

Apical membrane antigen 1 is a microneme protein which plays an indispensable role during Apicomplexa parasite invasion. The detailed mechanism of AMA-1 molecular interaction with its receptor on bovine erythrocytes has not been completely defined in *Babesia bovis*. This study was focused on identifying the minimum *B. bovis* AMA-1-derived regions governing specific and high-affinity binding to its target cells. Different approaches were used for detecting *ama-1* locus genetic variability and natural selection signatures. The binding properties of twelve highly conserved 20-residue-long peptides were evaluated using a sensitive and specific binding assay based on radio-iodination. *B. bovis* AMA-1 ectodomain structure was modelled and refined using molecular modelling software. NetMHCIIpan software was used for calculating B- and T-cell epitopes. The *B. bovis ama-1* gene had regions under functional constraint, having the highest negative selective pressure intensity in the Domain I encoding region. Interestingly, *B. bovis* AMA-1-DI (^100^YMQKFDIPRNHGSGIYVDLG^119^ and ^120^GYESVGSKSYRMPVGKCPVV^139^) and DII (^302^CPMHPVRDAIFGKWSGGSCV^321^)-derived peptides had high specificity interaction with erythrocytes and bound to a chymotrypsin and neuraminidase-treatment sensitive receptor. DI-derived peptides appear to be exposed on the protein’s surface and contain predicted B- and T-cell epitopes. These findings provide data (for the first-time) concerning *B. bovis* AMA-1 functional subunits which are important for establishing receptor-ligand interactions which could be used in synthetic vaccine development.

## 1. Introduction

*Babesia bovis* is an haemoprotozoan parasite which causes bovine babesiosis; it is transmitted by ticks from the genus Rhipicephalus, a vector which is distributed worldwide. This species is considered the most lethal agent from the genus Babesia since it causes severe clinical signs and high mortality rates in infected cattle, thereby representing a global menace for the livestock industry, involving huge annual financial losses worldwide [1,2]. Strategies have been developed for controlling the disease, such as using chemotherapeutics (against the parasite or vector) or vaccinating animals with attenuated parasites; such methods have not been very efficient given the increasing development of resistance to drugs and/or difficulties associated with attenuated vaccine production [3,4], highlighting the need for prioritising and improving control measures targeting this pathogen.

The need for making vaccines safer, more stable, and more effective has promoted their chemical development as an alternative strategy known today as a synthetic vaccine approach. This strategy has been widely applied on *Plasmodium falciparum*, mainly focusing on using a mixture of conserved peptides having binding properties derived from proteins which are critical for parasite survival [5]. Apical organelle molecules, such as micronemes and rhoptries, have been suitable candidate targets as some of them play an important role in invasion [6,7]. Apical membrane antígen-1 (AMA-1) is one of these, being a transmembrane microneme type I protein having an extracellular domain structured by 14 very conserved cysteine residues divided into 3 regions (I (DI), II (DII) and III (DIII) domains) [8].

Various studies have provided evidence that AMA-1 is essential for the formation of a multiprotein complex with rhoptry neck (RON) proteins, promoting the parasite’s strong binding to target cells, a highly conserved process within the phylum Apicomplexa [9,10,11,12,13]. Its functional importance is supported by the observation that its knockout is deleterious for Toxoplasma and Plasmodium parasites [14,15]. Interestingly, it has been demonstrated that *P. falciparum* AMA-1 (*Pf*AMA-1) contain poorly immunogenic high activity binding peptides (HABPs) to human erythrocytes which are very conserved and can inhibit parasite invasion in vitro [16]. However, replacing some *Pf*AMA-1 HABPs’ residues resulted in significantly increased immunogenicity, converting them into peptides able to trigger a powerful protection-inducing immune response in an animal model. This feature can be advantageous for developing a multi-component, subunit-based synthetic antimalarial vaccine [17,18].

AMA-1 proteins are expressed in most *Babesia* spp. analysed. AMA-1 has been identified in a lysate derived from *Babesia orientalis*-infected water buffalo blood [19]. In addition, it has been shown that the molecule is antigenic upon infection of dogs and cattle with *Babesia gibsoni* and *Babesia bigemina,* respectively [20,21]. *Babesia divergens-* and *Babesia microti-*derived AMA-1 bind to a receptor which is trypsin-sensitive for bovine erythrocytes and chymotrypsin sensitive for human ones [22,23]. Furthermore, AMA-1 can trigger the production of antibodies (Abs) capable of reducing *B. microti* and *Babesia ovis* invasion in vitro [22,24] and *B. bigemina* in vivo [25].

In *B. bovis*, AMA-1 is encoded by a single-copy single exon gene, encoding for a protein containing 605 amino acids. AMA-1 has been considered a potential vaccine candidate as it has limited polymorphism in strains from different geographical areas and can stimulate a response from the immune system of the host during natural infection [26]. It has been reported that Abs targeting a central DI-DII region (L193-T365) can reduce invasion by 70% and those targeting peptides located in DI (^46^FAFHREPTNRRLTRRA^60^) or DII (^395^RGVGMNWATYDKDSG^409^ and ^453^YVEPRAKTTNKYLDV^467^) by 65% [27,28]. However, despite significant progress on the characterization of the *B. bovis* AMA-1, it remains unknown which are the functionally relevant regions associated with erythrocyte receptor interaction. The identification of such regions is thus an additional strategy for finding suitable synthetic vaccine candidates.

A series of robust and highly sensitive experiments aiming at elucidating the minimum highly conserved regions governing *B. bovis* AMA-1 biding to bovine erythrocytes is here described. The identification of such regions, likely involved in the receptor-ligand interactions amongst the parasite and the erythrocytes of the bovine host, may aid in the design of synthetic vaccines.

## 2. Results

### 2.1. Ama-1 Has Regions Under Distinct Selective Pressure Intensity

The limited genetic variability and negative selection previously reported using *B. bovis* intraspecific data [26] supports the idea that *ama-1* may be subject to functional constraint. Phylogenetically sequences from closely-related Babesia species were used for testing natural selection to further investigate whether purifying selection has driven *ama-1* evolution. The McDonald-Kreitman (MK) test neutrality index (NI) was >1 for comparisons with *Babesia xinjiang* and *Babesia ovata*, indicating less nonsynonymous divergence than expected under neutrality. The complete gene’s *d_N_/d_S_* ratio was <1 for all comparisons, revealing fewer nonsynonymous than synonymous substitutions (Table 1). This outcome and the fact that bsREL analysis did not find evidence of episodic positive selection in any branch along the phylogeny analysed (Figure S1) suggested purifying selection acting on *ama-1*.

**Figure 1:**
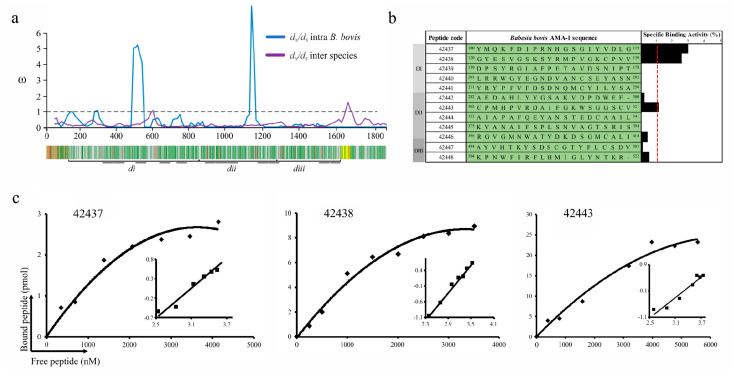
*B. bovis* AMA-1 peptide selection and binding to bovine erythrocytes. (**a**) The sliding window showing the ω rate (*y*-axis) and the nucleotide position (*x*-axis) (A ω < 1 means functional constraint). A gene diagram indicating the *B. bovis* AMA-1 encoding regions for the signal peptide (brown), the transmembrane region (yellow) and DI (di), DII (dii) and DIII (diii) can be observed below the sliding window. Negatively selected inter-species codons are shown in green and positively selected ones in red. (**b**) Binding profile for *B. bovis* AMA-1 domain-derived highly conserved peptides, carried out in triplicate. Cell binding activity percentage is represented by black bars. The binding activity (red line) was considered when each peptide had ≥1 binding percentage. (**c**) Peptide saturation curves. Log F = free peptide (*x*-axis) and log [B/bmax-B] (*y*-axis), where B = amount of bound peptide and Bmax = maximum amount of bound peptide, shown on the Hill plot (inset).

The *d_N_/d_S_* ratio was calculated considering that natural selection can act differentially on a protein’s regions; a sliding window was thus used to evaluate *d_N_/d_S_* behavior along the *ama-1* locus. Omega (ω) values between species were marginally >1 for the transmembrane region encoding region and close to 1 for a short fragment encoding DI (Figure 1a). Within *B. bovis,* ω values were >1 for two regions encoding DI and DII and close to 1 for those ones encoding the DI. Eighteen negatively and two positively selected sites were identified within *B. bovis*, whereas 425 negatively and 15 positively selected sites were found amongst babesial species. DI had the highest enrichment of negatively selected sites (90%; 172/190), followed by DII (74%; 104/139) and DIII (71%; 58/81) (Figure 1a). These results indicated that pervasive purifying selection has acted throughout *ama-1*, having neutral or positive selection sites located within DI and DII.

Conserved regions amongst Babesia AMA-1 homologue proteins and other Apicomplexan parasites known to invade red blood cells (RBC) (mainly *P. falciparum* and *Plasmodium vivax*) were found through profile-profile comparison using a sequence profile obtained from babesial sequence alignment (Figure S2). Besides intense negative selection signatures, these regions had identical residues (including two conserved cysteines, Cys136 and Cys302, forming a putative disulphide bond) or conservative amino acid (aa) replacements and secondary structure conservation being consistent with the observation that DI and DII were more conserved than DIII in Babesia [29] and that they had regions having high RBC binding activity in *P. falciparum* and *P. vivax* [10,30]. The forgoing, added to the finding regarding *B. bovis* AMA-1-DI-DII, suggested that functional constraint and purifying selection appear to have driven these regions’ evolution to conserve the sequence and structural features and could therefore be important for target cell binding activity.

### 2.2. B. bovis AMA-1 Bovine Erythrocyte Binding Activity is Mediated by Conserved Small Peptides

*B. bovis* AMA-1 specific binding to a bovine RBC receptor was ascertained by peptide-cell interaction competition assays. Fully conserved intra-species peptides whose encoding regions had ω < 1 were selected; 5 DI-, 5 DII- and 2 DIII-derived peptides were synthesised (Figure 1a). These synthetic peptides were radiolabelled and incubated with bovine erythrocytes in the presence or absence of the same unlabelled peptide to test their specificity and sensitivity binding properties. A specific binding slope plot from 0 to 0.03 was found when the peptides’ specific binding was calculated by subtracting non-specific binding from total binding (Figure 1b). Only DI-derived peptides 42437 (^100^YMQKFDIPRNHGSGIYVDLG^119^) and 42438 (^120^GYESVGSKSYRMPVGKCPVV^139^) and DII 42443 (^302^CPMHPVRDAIFGKWSGGSCV^321^) were considered HABPs considering a > 0.01 slope. Saturation analysis gave Kd = 1.1 μM and nH = 1.3 for peptide 42437, Kd = 1.3 μM and nH = 1.6 for 42438 and Kd = 2.4 μM and nH = 1.3 for 42443, with peptides located in DI being those that have the highest binding affinity (Figure 1c). Altogether, these data support the notion that AMA-1 DI is the protein region participating in the interaction with bovine erythrocytes mediated by two 20-residue-long peptides.

### 2.3. B. bovis AMA-1 HABPs Have High Affinity Biding to Enzyme Treatment-Sensitive Receptors

**Figure 2:**
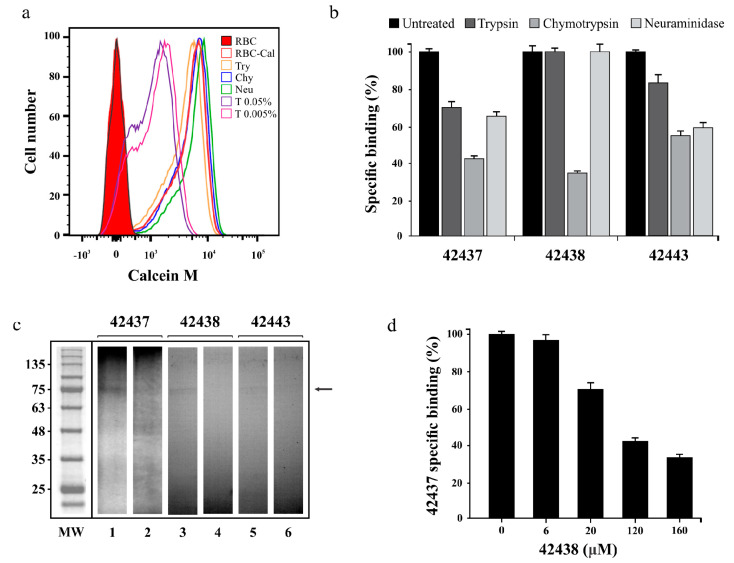
Peptides’ specific binding activity. (**a**) Cell viability assay showing Calcein M signal (*x*-axis) and the amount of cells (*y*-axis). Bovine RBC treated with trypsin (Try) chymotrypsin (Chy), neuraminidase (Neu), Triton X-100 (T) or dyed with Calcein M (RBC-Cal) are also shown. (**b**) Specific binding effect of enzyme treatment on *B. bovis* AMA-1 42437, 42438 and 42443 peptides. The specific enzyme-treated erythrocyte binding percentage is represented by a bar. Untreated RBC were used as control. (**c**) Cross-linking assay. The autoradiogram shows radiolabelled HABPs’ total (odd lane) and inhibited (even lane) binding to erythrocytes using non-radiolabelled peptides. The molecular weight (MW) pattern and receptor (arrow) found are also shown. (**d**) Peptide 43437 binding competition assay, using different non-radiolabelled peptide 42443 concentrations. The 42443 binding percentage and μM concentration are shown. Standard deviations for Figure 2b,d were below 5%.

Binding assays involving trypsin-, chymotrypsin-, and neuraminidase-treated cells were done for determining the specific binding properties of *B. bovis* AMA-1 HABPs to a bovine erythrocyte receptor. Calcein M led to confirming that no enzyme treatment affected erythrocyte viability as more than 99% of the cells were viable, unlike those treated with triton X-100, which caused 25% of cell lysis (cell mortality control) (Figure 2a). Analysis of peptide 42437 binding profile revealed that it became reduced by 58% with chymotrypsin-treated erythrocytes, 36% for neuraminidase-treated, and 31% for trypsin-treated ones (Figure 2b). Peptide 42438 interaction with chymotrypsin-treated cells became reduced by 66%, whilst there was no effect using trypsin- and/or neuraminidase-treated cells (Figure 2b). Peptide 42443 interaction with bovine erythrocytes was affected by 46% using chymotrypsin treatment and 42% with neuraminidase, whilst there was a lesser effect with trypsin treatment (Figure 2b).

A cross-linking assay was carried out to confirm the binding specificity and approximate molecular weight of the receptor with which *B. bovis* AMA-1 peptides 42437, 42438, and 42443 interacted. It was found that the three peptides were capable of specifically binding to a receptor having an apparent 75 kDa molecular weight as the intensity of the radioactive signal became reduced when an excess of the same non-radiolabelled peptide was used; such interaction was significantly less intense for peptide 42443 (Figure 2c). A cross-competition assay was carried out as peptides 42437 and 42438 were adjacent, finding that non-radiolabelled peptide 42438 competed with peptide 42437 in a concentration-dependent manner (Figure 2d); this supports the idea that peptides are bound to the same receptor site or a very close region, thereby confirming the DI region’s functionality.

### 2.4. B. bovis AMA-1 HABPs 42437 and 42438 are Promising Synthetic Vaccine Candidates

**Figure 3:**
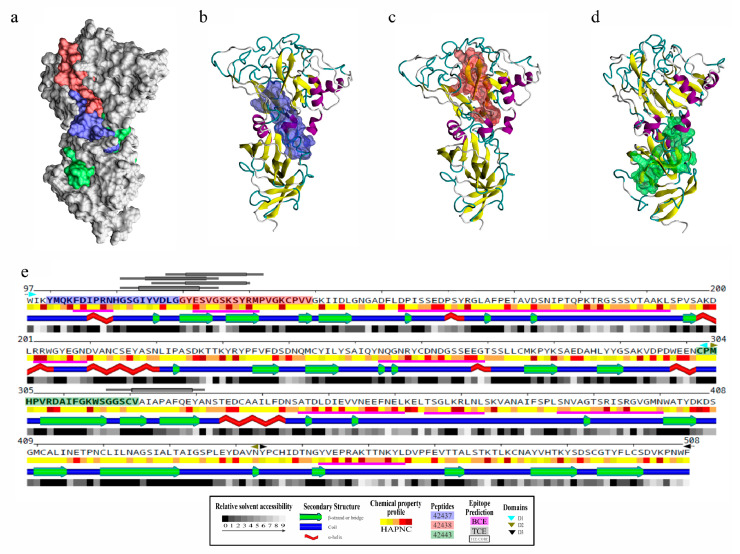
Structural analysis and B- and T-cell epitope prediction for *B. bovis* AMA-1-DI-II. (**a**) Localisation on the surface view of HABPs 42437 (blue), 42438 (red), and 42443 (green). Peptides 42437 (**b**) and 42438 (**c**) had more surface-accessible residues than peptide 42443 (**d**). (**e**) Secondary structure representation, including the relative solvent accessibility and physical-chemical amino acid profile. Peptide sequences are presented in bold and boxed using the same color code as in panel a. DI (cyan triangles), DII (green triangles), and DIII (black triangles) are also indicated. Regions containing B-cell epitopes are highlighted by magenta bars and T-cell ones by grey bars.

The ectodomain was structurally modelled to analyse each peptide’s spatial location since it was found that *B. bovis* AMA-1 had HABPs to target cells. The methodology enabled obtaining very good quality 3D models (Figure S3a); four missing loops in the *B. divergens* structure were modelled ab-initio to complete the *B. bovis* AMA-1 homology model (Figure S3b). It was observed that peptides 42437 and 42438 had more significantly surface-exposed sectors than 42443 when *B. bovis* AMA-1 HABPs were located on 3D structure (Figure 3a–d), supported by solvent accessibility analysis (Figure 3e). This finding agreed with the highest binding activity and affinity found for peptides 42437 and 42438 (Figure 1b,c), indicating that they could be involved in target cell receptor binding.

B- and T-cell epitopes on *B. bovis* AMA-1 protein were predicted as immune control against this pathogen relies mainly on humoral and cellular responses [31,32,33]. It was observed that modelled loops had most predicted B-cell epitopes and, particularly interesting, the region formed by peptides 42437 and 42438 had two sequential B-cell epitopes and three T-cell epitopes (one having two alternative binding frames) predicted as strong binders for multiple BoLA-DR molecules, unlike peptide 42443, which only had an adjacent T-cell epitope having an affinity spectrum for fewer alleles (Figure 3e, Table S1). This result led to inferring that the 42437 and 42438 peptides could be regions of interest for the design of novel interventions aimed at blocking bovine erythrocyte invasion by *B. bovis* parasites.

## 3. Discussion

Discovering receptor-ligand interactions occurring between parasite molecules and those of their target cells (more specifically, the minimum interacting regions) is a promising strategy for developing effective control methods against microorganisms. So far, only the role of TRAP2 [34] and RAP1 [35] proteins binding to *B. bovis* bovine erythrocytes has been described; however, no study to date has reported specific target cell binding regions for any molecule. AMA-1 has been one of the most studied proteins, based on functional evidence reported in Apicomplexa microorganisms invading erythrocytes [14,15]. This molecule has specialised regions able to establish inter- (with erythrocyte receptors) [16] and intra- (with RON proteins) [9,13] molecular interactions that may facilitate adhesion and invasion to their target cells. Considering that inter-molecular interactions are important for vaccine development [5], this work was thus designed towards identifying the minimum regions participating in *B. bovis* AMA-1 binding to bovine erythrocytes.

It has been reported that functional or structurally important regions within a protein tend to evolve much more slowly than those which are not constrained by functionality [36]; these are usually conserved amongst related species and have intense purifying selection signatures [37]. Natural selection action on *ama-1* using a sequence dataset of phylogenetically closely-related Babesia and *B. bovis* species were analysed for identifying regions under functional constraint since *ama-1* has been shown to have limited variability and negative selection signatures within *B. bovis* [26]. Such analysis led to finding that *ama-1* had intense negative selection signals in babesial species with no evidence of lineages having episodic positive selection (Figure S1). McDonald–Kreitman test results and the *d_N_/d_S_* ratio for *B. bovis* and closely-related species (*B. orientalis*, *B. xinjiang*, *B. bigemina*, *B. ovata* and *B. divergens*) suggested that *ama-1* could be under intense functional constraint, similar to that reported for other Apicomplexan parasites [38]. Limited variability and purifying selection signatures were thus identified, indicating functional constraint on regions within *B. bovis ama-1*. It is worth highlighting that negative selection signatures were also detected in almost all *ama-1* loci when *d_N_/d_S_* ratio was evaluated using a sliding window, thus agreeing with results for the whole gene (Figure 1a). Analysis indicated that AMA-1 might have functionally relevant regions which could be explored to evaluate their role in target cell adhesion-related *B. bovis* biology.

Twelve highly conserved *B. bovis* AMA-1 20-residue-long regions under negative selection were thus selected to find which of them were involved in bovine erythrocyte binding based on a sensitive and specific binding assay extensively used for detecting protein’s highly conserved functional regions involved in the interaction with *P. falciparum*, *P. vivax* and *Mycobacterium tuberculosis* target cells [5,10,39]. The assays showed conclusively that two *B. bovis* AMA-1 DI-derived peptides (42437 and 42438) and one from DII (42443) bound specifically to erythrocytes. In addition, peptides 42437 and 42438 displayed low affinity constants, indicating positive cooperativity (Figure 1b,c). No DIII-derived peptide had binding activity consistent with results for *P. vivax* (*Pv*AMA-1) and *Plasmodium yoelii* AMA-1 (*Py*AMA-1), given that such regions do not take place in erythrocyte binding [10,40]. It has been shown that Abs directed against some peptides located in *B. bovis* AMA-1 DI (^46^FAFHREPTNRRLTRRA^60^) and DII (^395^RGVGMNWATYDKDSG^409^ and ^453^YVEPRAKTTNKYLDV^467^) were able to reduce invasion by 65% [27]. DII-derived ^395^R-G^409^ did not interact with bovine erythrocytes whilst ^46^F-A^60^ and ^453^Y-V^467^ peptides were not considered for peptide-cell interaction assays given their polymorphic nature (Figure 1b). Due to the above and given that a suitable vaccine candidate must be involved in protein-receptor interaction and avoid an allele-specific immune response [41], it could be suggested that peptides 42437, 42438, and 42443 are promising candidates for evaluating their usefulness as synthetic vaccine components.

It was found that binding was significantly reduced by chymotrypsin treatment for the three HABPs, and by sialic acid residue excision when using neuraminidase for peptides 42437 and 42443 (Figure 2b,c). This profile was similar to that reported for the HABP found in *Pv*AMA-1-DI-II (21270) but different for recombinant *Pf*AMA-1-DIII (trypsin sensitive and sialic acid independent) [42] and *B. divergens* and *B. microti* native AMA-1 (trypsin and chymotrypsin sensitive but neuraminidases resistant) [22,23], suggesting that AMA-1 could interact with distinct receptors depending on the target cell, despite being conserved within the Apicomplexa phylum. Interestingly, it has also been reported that *B. bovis* invasion in vitro is mainly affected by neuraminidase treatment (77.5 ± 2.5% inhibition), followed by chymotrypsin (33 ± 7.7% inhibition) and trypsin (26 ± 2.2% inhibition) [43]. The erythrocyte invasion process is complex and involves at least an initial step of recognition, following the re-orientation and formation of a tight junction between the parasite and the erythrocyte. All these steps require a coordinated secretion of proteins derived from the apical organelles, AMA-1, being secreted to establish the tight junction [9,13]. It is thus possible that although *B. bovis* invasion to bovine erythrocytes could mainly be sialic acid residue-mediated, the parasite attachment to cells through AMA-1 occurs with a protein nature receptor just before tight junction formation. This is consistent with the AMA-1 peptides’ enzyme profile given that some of them could interact with the receptor’s backbone whilst others interacted with sialic acid residues (Figure 2b). Therefore, and taking into account the reported role of glycophorins A and B in *Babesia* invasion [44,45], it can be suggested that the *B. bovis* AMA-1 receptor is a sialoglycoprotein; however, further assays are required to confirm such hypothesis.

*B. bovis* AMA-1 target cell HABPs were compared to those from other Apicomplexan parasites known to invade RBC, such as *P. falciparum* and *P. vivax* (Figure S2). Interestingly, peptide 42438 had 61.9% similarity with a HABP found in *P. falciparum* (4313: ^134^DAEVAGTQYRLPSGKCPVFG^153^) and 43.5% for that found in *P. vivax* (21270: ^81^EVENAKYRIPAGRCPVFGKG^100^) whilst peptide 42443 had 55% similarity with that found in *Pf*AMA-1 (consisting of HABP 4321- (^294^VVDNWEKVCPRKNLQNAKFGY^313^) and 4322- (^314^LWVDGNCEDIPHVNEFSAIDY^333^) derived regions). It has been reported that peptide 4313 was able to inhibit *P. falciparum* invasion of erythrocytes by 72% [16] whilst peptide 21270 inhibited recombinant *Pv*AMA-1-DI-II interaction with human reticulocytes [10]. Such finding supported the idea that *B. bovis* AMA-1-derived 42438 peptide could play a critical role in target cell binding.

Since *B. bovis* AMA-1 have failed to stimulate protective immunity [27,28], identifying novel highly conserved regions having the potential to be B- or T-cell epitopes may prove important for effective vaccine development. Interestingly, the DI region where the 42437 and 42438 peptides are located (Figure 3a-d) had two regions which could be B-cell epitopes and another three having high potential to be processed and presented to several BoLA-DR molecules (Figure 3e). It has been suggested that immune control against this pathogen relies mainly on MHC class II–restricted CD4+ T-helper cell responses due to the lack of a nucleated cell stage in the *B. bovis* lifecycle in the vertebrate host [31,33]; antigens inducing this type of response can activate macrophages and stimulate the production of neutralising and opsonising antibodies (nAbs) in cattle, thus protecting against *B. bovis* challenge. Characteristics found in peptides 42437 and 42438 regarding their conservation and selective landscape, as well as their binding properties and potential B- and T-cell epitope regions highlight them as potential candidates for the development of a synthetic vaccine against *B. bovis*. Future studies evaluating the peptides’ ability to induce a protective immune response could contribute towards confirming such hypothesis.

## 4. Materials and Methods

### 4.1. Predicting B. bovis Ama-1 Functional Constraint Regions

Nucleotide sequences from *B. bovis* isolates from Thailand: Chiang Mai (accession numbers KY575955, KY575956, KY575957), Sri Lanka: Polonnaruwa, Ampara and Jaffna (AB787632, AB787633, AB787634, AB787635, AB787636, AB787637), Israel: Bet-Dagan (AY486101, KX196262, KX196263), Brazil: Northeast, North, Southeast, South and Midwest regions (FJ588024, FJ588025, FJ588026, FJ588027, FJ588028) and the United States: MD, Rockville (XM_001610993), along with phylogenetically close species *B. orientalis* (KJ196379), *B. xinjiang* (XP_028870051), *B. bigemina* (PiroplasmaDB ID: BBBOND_0109200), *B. ovata* (PiroplasmaDB ID: BOVATA_017080) and *B. divergens* (PiroplasmaDB ID: Bdiv_023990c), were retrieved from the NCBI GenBank [46] or PiroplasmaDB sequence databases (Release 46 6 Nov 2019) [47]. Deduced aa sequences were aligned using MUSCLE [48] and PROMALS3D [49] multiple sequence and structure alignment servers; The MergeAlign algorithm [50] was used for constructing a consensus alignment which was manually adjusted, based on profile-profile comparisons and secondary and tertiary structure information. The HHpred server [51] was used for profile-profile comparisons and TranslatorX [52] was used for obtaining final codon alignment.

Several methods were used for evaluating natural selection action on the *ama-1* locus. The McDonald–Kreitman test was used for comparing polymorphism and divergence patterns, using a web server [53]. MEGA X [54] was used for calculating the ratio of nonsynonymous substitutions per nonsynonymous site to synonymous substitutions per synonymous site (*d_N_/d_S_*) with the modified Nei-Gojobori method and Jukes-Cantor correction, using the Z-test for evaluating significance. DnaSP v.6 [55] DNA polymorphism analysis software was used for calculating the *d_N_/d_S_* ratio regarding alignments using a sliding window with the Nei-Gojobori method and Jukes-Cantor correction [56]. Phylogeny was inferred by maximum likelihood using the MEGA X sequence alignment tool [54], showing that the sequence dataset was divergent enough for confidently evaluating natural selection signals (8.1 expected substitutions per codon along the tree and 0.9 substitutions per codon average branch length) [57]. The Datamonkey web server [58] was used for identifying lineages under episodic diversifying selection (Branch-site REL method [59]) and potential recombination breakpoints (GARD method). Codons under negative and positive selection were inferred using maximum likelihood (FEL, SLAC, REL [60], FUBAR [61]) and Bayesian methods (MEME [62]), taking <0.1 *p*-value (FEL, SLAC and MEME), >0.9 posterior probability (FUBAR) or >50 Bayes factor (REL) as significant.

### 4.2. B. bovis AMA-1 Peptide Selection, Synthesis and Radiolabelling

Peptide selection was based on the degree of conservation and the presence of functionally constricted regions found throughout *B. bovis ama-1* gene. Twelve peptides were synthesised using the tert-butoxycarbonyl (t-Boc) strategy and solid-phase synthesis methodology [63]. Cysteine residues were replaced by serine residue to avoid oxidation; a tyrosine residue was added to peptides lacking one in their native sequences to enable radiolabelling. The peptides were analysed using reversed-phase high-performance liquid chromatography (RP-HPLC) and MALDI-TOF mass spectrometry (Bruker Daltonics) after having been cleaved by the low–high hydrogen fluoride technique. Every peptide (10 μL) diluted at 1mg/mL in 4-(2-hydroxyethyl)-1-piperazineethanesulphonic acid buffered saline (HBS) was individually radiolabelled for 15 min using 3 μL Na^125^I (100 mCi/mL; ARC) and 15 μL chloramine T (2.75 mg/mL). After halting the reaction with 15 μL sodium metabisulfite (2.25 mg/mL), each peptide was purified by size exclusion chromatography using Sephadex G-10 columns (Pharmacia). A gamma counter (Packard Cobra II) was used for measuring/quantifying single samples’ radioactivity.

### 4.3. Blood Collection and Animal Handling

A blood sample was obtained by jugular venepuncture from a clinically healthy Normande 18month-old bull and collected in anticoagulant tubes. The animal was properly restrained/fastened and then its head was secured by a strap and rope. After locating the external jugular vein and applying pressure, the area was disinfected with ethanol for inserting the needle at a 30° angle and 20 mL blood samples were collected in tubes containing sodium citrate using the closed system. The above procedures and animal maintenance and handling were carried out at the Remanso farm, according to guidelines for the care of large animals established by Universidad de Ciencias Aplicadas y Ambientales (U.D.C.A)’s ethics committee (regulated by agreement No. 285/2008) using the protocol described in the minute 201901. The collected blood was washed thrice with HBS (1:1 ratio) to eliminate the sera and the leucocyte layer by spinning at 1600× *g* for 5 min for each run. A 60% erythrocyte solution was incubated at 37 °C for 1h 30 min with trypsin (1 mg/mL), chymotrypsin (1 mg/mL) and neuraminidase (100 μU/mL). After three washes using 250× *g* for 3 min, 4 × 10^5^ erythrocytes were incubated with 5 μM Calcein M at 37 °C for 45 min in the dark. An assay was carried out with erythrocytes treated at 37 °C for 30 min or with 0.05% or 0.005% triton X-100 for 15 min at room temperature (RT) for using them as cell lysis controls. A FACSC Canto II cytometer (BD) was used for determining cell viability by analysing 100,000 events.

### 4.4. Radiolabelled Peptide Bovine Erythrocyte Binding Assays and Dissociation Constants

The assays for every *B. bovis* AMA-1-derived synthetic peptide binding to bovine erythrocytes were carried out as described for *P. falciparum,* with some modifications [10]. Briefly, initial binding was screened in triplicate by incubating 7.5 × 10^7^ normal cells for 2 h at RT with increasing concentrations of each radiolabelled peptide (20–200 nM) in the absence (total binding) or presence (nonspecific binding) of the same non-radiolabelled peptide (20 μM) at 200 μL final volume. A similar binding assay was done with enzymatically-treated cells for 1 h at 37 °C without shaking. HABP bovine erythrocyte binding selection criteria were determined according to that established for *P. falciparum* [30]. Briefly, the Ka.r = [b]/[l] equation was used, where Ka is the association constant, r the receptor sites, b bound ligand and l free ligand. The [b]/[l] ratio is the binding activity, represented by the slope (m) of the specific binding curve. Peptides having a specific binding curve greater than or equal to 0.01 (1%) (0.010pmol bound peptide/pmol added peptide) were considered HABPs.

### 4.5. Saturation Assays

Modified binding assays were carried out to determine kinetic constants associated with *B. bovis* AMA-1 HABP binding interaction with bovine RBCs. Briefly, 7.5 × 10^7^ RBCs were incubated with a wide range of concentrations of each radiolabelled HABP (0–6000 nM) in the absence or presence of unlabelled peptide (~26 μM) at final 245 μL volume. Samples were incubated for 90 min at RT and then washed twice with HBS before measuring cell-associated radioactivity on a gamma counter. Equilibrium dissociation constants (kD), equivalent to the ligand concentration (moles/litre) occupying half of the receptors, were calculated by equilibrium phase curve (using a 1:1 interaction model). Hill coefficients (nH) were calculated by taking saturation results; positive cooperativity was assumed if nH had values higher than 1 which could have been the result of a binding site’s increased affinity due to a ligand’s previous binding to another site. Conversely, nH values lower than 1 would have indicated negative cooperativity (also called antagonism) and the first ligand molecule’s binding would have reduced the probability of a second molecule binding [64].

### 4.6. Receptor Identification by Cross-Linking

Cross-linking assays were used in triplicate for evaluating *B. bovis* AMA-1 interaction with any membrane receptor. The assay consisted of incubating radiolabelled peptide with 7.5 × 10^7^ bovine erythrocytes (*v*/*v*) at RT for 2 h at 4 rpm. A competition assay was used as control using an excess of non-radiolabelled peptide. The mixture was incubated for two hours at RT with 0.4 µg/mL bis(sulphosuccinimidyl) suberate (BS3) and then washed thrice with HBS. The cells were then lysed in 30 µL lysis buffer (15 µL buffer A (50 mM Tris-HCl, 100 mM NaCl and 0.1 mM EDTA) and 15 µL buffer B (0.5 M Tris-HCl, 10% SDS (*w*/*v*), 25% glycerol, 0.5% (*w*/*v*) bromophenol blue and 50μL β-mercaptoethanol, pH 6.8)) and spun at 14,000× *g* for 10 min. SDS-PAGE was used for separating the proteins and radioactivity signals were observed by autoradiography. Radiolabelled peptide 43437 (1 µM) binding competition assay involved using different non-radiolabelled peptide 42438 concentrations (0–160 µM).

### 4.7. B. bovis AMA-1 Ectoplasmic Region Structural Modelling and B- and T-Cell Epitope Prediction

MODELLER 9.25 protein structure modelling software [65,66,67] was used for modelling undetermined loops and refining them in the available crystallographic structure for the *B. divergens* ectoplasmic region (PDB:4APM). NAMD 2.12 parallel molecular dynamics software using CHARMM36 protein force field [68] and an isothermal/isobaric assembly (P = 1 atm, T = 273K) with the TIP3 solvation model [69] were used for refining the structure by 0.25 ns energy minimisation, followed by 1.0 ns molecular dynamics and 0.25 final minimisation. SCWRL4 protein side-chain conformation prediction software [70] was used for making the necessary substitutions in this structure to create a *B. bovis* AMA-1 ectodomain model, using the T2Bo sequence (XM_001610993.1) as reference (see alignment in Figure S3, panel b). The same refinement procedure described for *B. divergens* was then used. The models’ quality was evaluated using MOLPROBITY structure-validation software and Qualitative Model Energy ANalysis (QMEAN4) scores [71,72]. The final model for each molecule was visualised using VMD 1.9.3 molecular visualisation software [73] to detect *B. bovis* AMA-1 peptides having higher than 1% binding. PolyView image viewer and editor [74] was used for calculating each aa’s secondary structure annotation, physical-chemical profile and relative accessibility to solvent.

The BepiPred-2.0 [75] server was used for predicting B-cell epitopes, using a 0.56 epitope threshold. NetMHCIIpan 4.0 software [76] was used for predicting T-cell epitopes, using the context encoding option for predicting peptide elution affinities for BoLA-DR molecules. An *ad-hoc* version of the predictor was used for estimating peptide binding for previously reported 135 BoLA-DR molecules [77]. The percentile rank distribution for each peptide size/BoLA-DR was produced using a set of 200,000 non-redundant random UnitProtKB/Swiss-Prot derived peptides [78]. The threshold for a strong binder was ≤2% rank and >2% to ≤10% rank for weak binders.

## 5. Conclusions

Novel findings on *B. bovis* AMA-1 minimum regions participating in bovine erythrocyte interaction have been reported for the first time in this study. *B. bovis* AMA-1 has three regions (peptides 42437, 42438 and 42443) under selective pressure which participate in high-affinity binding to erythrocytes via a receptor containing sialic acid residues. Given that peptides 42437 and 42438 were highly conserved amongst *B. bovis* strains, had bovine erythrocyte binding properties, were surface exposed and consisted of B- and T-cell epitopes, they are attractive for designing a synthetic vaccine against *B. bovis*. Future work should be focused on evaluating 42437 and 42438 Abs capability for blocking *B. bovis* AMA-1 binding to bovine erythrocytes and their correlation in in vitro invasion inhibition assays.

## Figures and Tables

**Table 1:** Natural selection on *ama-1* locus.

	MK		*d_N_/d_S_*			
Babesia Species	NI	*p*-Value	*d_N_*	*d_S_*	*d_N_/d_S_*	*p*-Value
*B. orientalis*	1.131	0.617	0.0356	0.1908	0.187	<0.001
*B. xinjiang*	1.519	0.046	0.0465	0.1864	0.250	<0.001
*B. bigemina*	1.317	0.273	0.0591	0.1986	0.298	<0.001
*B. ovata*	2.136	0.004	0.0583	0.1973	0.295	<0.001
*B. divergens*	1.216	0.341	0.063	0.1933	0.326	<0.001

MK: the McDonald–Kreitman neutrality index (NI) regarding the pairwise comparison amongst *B. bovis* and phylogenetically-related species. *d_N_/d_S_*: the ratio of the amount of nonsynonymous substitutions per nonsynonymous site to the amount of synonymous substitutions per synonymous site.

## Data Availability

The data presented in this study are available within the article and in the supplementary material.

## Supplementary Materials

The following are available online at https://www.mdpi.com/1422-0067/22/2/714/s1.

## Abbreviations

AMA-1	apical membrane antigen 1
RON	rhoptry neck protein
TRAP	thrombospondin-related anonymous protein
RAP1	rhoptry-associated protein 1
HABPs	high activity binding peptides
RBC	red blood cells
D	domain
