# Supplementary material for: Babesia Bovis Ligand-Receptor Interaction: AMA-1 Contains Small Regions Governing Bovine Erythrocyte Binding
Source: Int J Mol Sci. 2021 Jan 13;22(2):714. doi: 10.3390/ijms22020714 (PMC7828228; doi:10.3390/ijms22020714)
Supplement: Supplementary file 1 [file ijms-22-00714-s001.pdf]

International Journal of *Molecular Sciences*

1. Supplementary files

Supplementary Figure 1. Lineages under episodic positive selection identified by the branch site REL method.

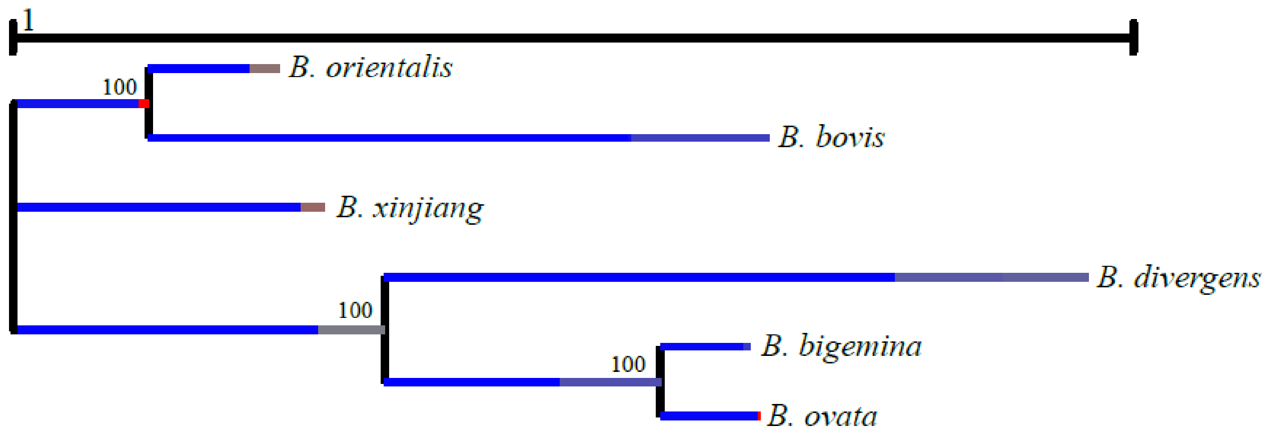

Phylogeny was inferred by using the MEGA X sequence alignment tool, along with the maximum likelihood method, using the K2P+G (5) evolutionary model and was scaled on the expected amount of substitutions/nucleotides. Each colour's hue indicates selection strength, primary red being ω >5, primary blue ω=0 and grey ω=1.

Supplementary Figure 2. Sequence and secondary structure conservation of two AMA-1 regions derived from Babesia and Plasmodium species.

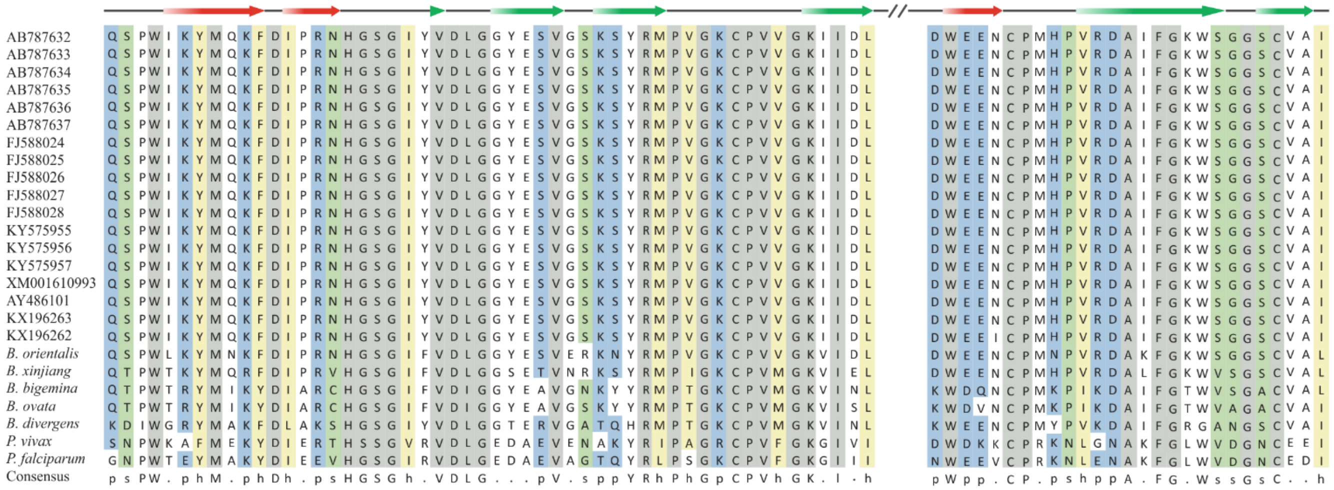

B. bovis sequences are designated by their accession numbers. Red arrows above the alignment represent α-helices and green ones β-sheets/turns; polar (p), small (s) and hydrophobic (h) residues are indicated on the consensus sequence.

International Journal of *Molecular Sciences*

MDPI

Supplementary Figure 3. *B. bovis* AMA-1 ectoplasmic domain structure refinement.

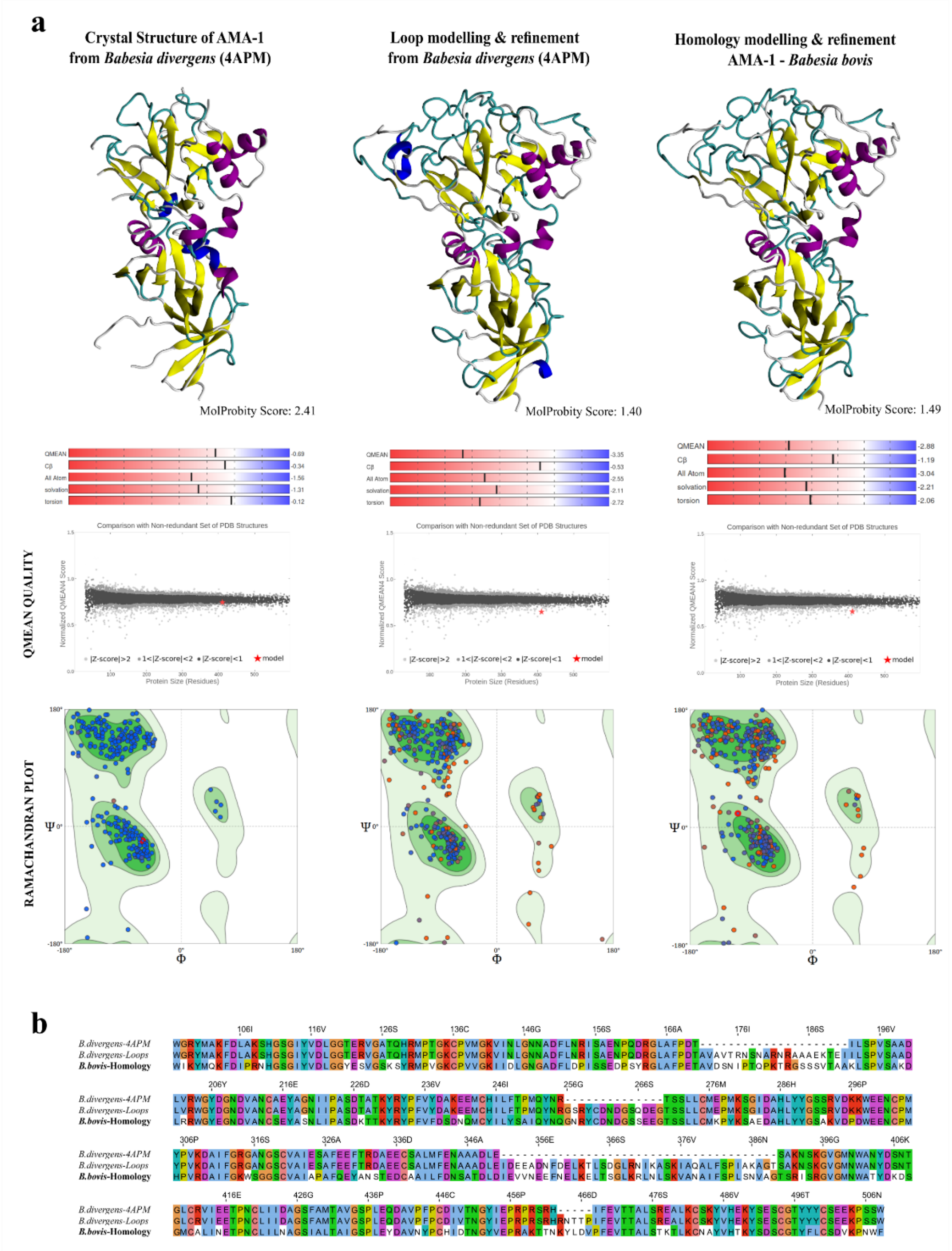

(**a**) Babesia AMA-1 modelling structures and quality parameters. MolProbity structure validation score (combined protein quality score reflecting the crystallographic resolution at which such quality would be expected, a lower score being better). QMEAN4 is a scoring function calculated by comparison with reference structures and ranges from -4 to 4; the smaller the value, the worse a model's quality. Normalised QMEAN4, comparing the models studied here with reported crystallographic structures. Note how the homology model was close to acceptable structural parameters. The models had good

Ramachandran map profiles, with few outliers. (**b**). AMA-1 sequence alignment regarding the structures studied here.

Supplementary Table 1. *B. bovis* AMA-1-DI-DII HABP T-cell epitopes.

Start position	Allele	Peptide (15 mer)	Core (P1-P9)	Rank-EL	*B. bovis* AMA-1 HABP
111	BoLA-DRB3_010_02	**GSGIYVDLGGYESVG**	YVDLGGYES	1.22	**42437** / **42438**
111	BoLA-DRB3_020_02	**GSGIYVDLGGYESVG**	YVDLGGYES	1.26	**42437** / **42438**
111	BoLA-DRB3_060_01	**GSGIYVDLGGYESVG**	YVDLGGYES	1.50	**42437** / **42438**
111	BoLA-DRB3_031_01	**GSGIYVDLGGYESVG**	IYVDLGGYE	1.65	**42437** / **42438**
111	BoLA-DRB3_031_03	**GSGIYVDLGGYESVG**	IYVDLGGYE	1.65	**42437** / **42438**
111	BoLA-DRB3_015_06	**GSGIYVDLGGYESVG**	IYVDLGGYE	1.66	**42437** / **42438**
111	BoLA-DRB3_043_03	**GSGIYVDLGGYESVG**	YVDLGGYES	1.68	**42437** / **42438**
111	BoLA-DRB3_033_01	**GSGIYVDLGGYESVG**	IYVDLGGYE	1.70	**42437** / **42438**
111	BoLA-DRB3_043_01	**GSGIYVDLGGYESVG**	IYVDLGGYE	1.79	**42437** / **42438**
111	BoLA-DRB3_066_01	**GSGIYVDLGGYESVG**	YVDLGGYES	1.79	**42437** / **42438**
111	BoLA-DRB3_027_04	**GSGIYVDLGGYESVG**	YVDLGGYES	1.85	**42437** / **42438**
111	BoLA-DRB3_007_02	**GSGIYVDLGGYESVG**	YVDLGGYES	1.88	**42437** / **42438**
111	BoLA-DRB3_027_06	**GSGIYVDLGGYESVG**	IYVDLGGYE	1.90	**42437** / **42438**
111	BoLA-DRB3_005_04	**GSGIYVDLGGYESVG**	IYVDLGGYE	1.93	**42437** / **42438**
111	BoLA-DRB3_043_02	**GSGIYVDLGGYESVG**	IYVDLGGYE	1.96	**42437** / **42438**
111	BoLA-DRB3_014_01_01	**GSGIYVDLGGYESVG**	IYVDLGGYE	1.97	**42437** / **42438**
111	BoLA-DRB3_014_01_02	**GSGIYVDLGGYESVG**	IYVDLGGYE	1.97	**42437** / **42438**
116	BoLA-DRB3_028_01	**VDLGGYESVGSKSYR**	YESVGSKSY	0.75	**42437** / **42438**
116	BoLA-DRB3_028_02	**VDLGGYESVGSKSYR**	YESVGSKSY	0.75	**42437** / **42438**
116	BoLA-DRB3_011_01	**VDLGGYESVGSKSYR**	YESVGSKSY	1.24	**42437** / **42438**
116	BoLA-DRB3_028_03	**VDLGGYESVGSKSYR**	YESVGSKSY	1.25	**42437** / **42438**
116	BoLA-DRB3_031_04	**VDLGGYESVGSKSYR**	YESVGSKSY	1.27	**42437** / **42438**
116	BoLA-DRB3_075_03	**VDLGGYESVGSKSYR**	YESVGSKSY	1.28	**42437** / **42438**
116	BoLA-DRB3_022_03	**VDLGGYESVGSKSYR**	YESVGSKSY	1.36	**42437** / **42438**
116	BoLA-DRB3_075_02	**VDLGGYESVGSKSYR**	YESVGSKSY	1.37	**42437** / **42438**
116	BoLA-DRB3_010_02	**VDLGGYESVGSKSYR**	YESVGSKSY	1.40	**42437** / **42438**
116	BoLA-DRB3_066_01	**VDLGGYESVGSKSYR**	YESVGSKSY	1.41	**42437** / **42438**
116	BoLA-DRB3_060_01	**VDLGGYESVGSKSYR**	YESVGSKSY	1.47	**42437** / **42438**
116	BoLA-DRB3_022_01	**VDLGGYESVGSKSYR**	YESVGSKSY	1.51	**42437** / **42438**
116	BoLA-DRB3_022_02	**VDLGGYESVGSKSYR**	YESVGSKSY	1.51	**42437** / **42438**
116	BoLA-DRB3_022_04	**VDLGGYESVGSKSYR**	YESVGSKSY	1.51	**42437** / **42438**
116	BoLA-DRB3_022_05	**VDLGGYESVGSKSYR**	YESVGSKSY	1.51	**42437** / **42438**
116	BoLA-DRB3_026_01	**VDLGGYESVGSKSYR**	YESVGSKSY	1.56	**42437** / **42438**
116	BoLA-DRB3_007_02	**VDLGGYESVGSKSYR**	YESVGSKSY	1.57	**42437** / **42438**
116	BoLA-DRB3_006_01	**VDLGGYESVGSKSYR**	YESVGSKSY	1.63	**42437** / **42438**

International Journal of *Molecular Sciences*

116	BoLA-DRB3_074_01	**VDLGGYESVGSKSYR**	YESVGSKSY	1.63	**42437** / **42438**
116	BoLA-DRB3_007_01	**VDLGGYESVGSKSYR**	YESVGSKSY	1.66	**42437** / **42438**
116	BoLA-DRB3_043_01	**VDLGGYESVGSKSYR**	YESVGSKSY	1.75	**42437** / **42438**
116	BoLA-DRB3_075_01	**VDLGGYESVGSKSYR**	YESVGSKSY	1.76	**42437** / **42438**
116	BoLA-DRB3_031_01	**VDLGGYESVGSKSYR**	YESVGSKSY	1.86	**42437** / **42438**
116	BoLA-DRB3_031_03	**VDLGGYESVGSKSYR**	YESVGSKSY	1.86	**42437** / **42438**
116	BoLA-DRB3_010_01	**VDLGGYESVGSKSYR**	YESVGSKSY	1.99	**42437** / **42438**
118	BoLA-DRB3_028_01	**LGGYESVGSKSYRMP**	YESVGSKSY	1.24	**42437** / **42438**
118	BoLA-DRB3_028_02	**LGGYESVGSKSYRMP**	YESVGSKSY	1.24	**42437** / **42438**
118	BoLA-DRB3_043_01	**LGGYESVGSKSYRMP**	YESVGSKSY	1.73	**42437** / **42438**
118	BoLA-DRB3_066_01	**LGGYESVGSKSYRMP**	YESVGSKSY	1.91	**42437** / **42438**
118	BoLA-DRB3_043_02	**LGGYESVGSKSYRMP**	YESVGSKSY	1.94	**42437** / **42438**
317	BoLA-DRB3_022_03	GGSCVAIAPAFQEYA	VAIAPAFQE	1.43	42443
317	BoLA-DRB3_028_03	GGSCVAIAPAFQEYA	VAIAPAFQE	1.50	42443
317	BoLA-DRB3_011_01	GGSCVAIAPAFQEYA	VAIAPAFQE	1.62	42443
317	BoLA-DRB3_014_01_01	GGSCVAIAPAFQEYA	VAIAPAFQE	1.72	42443
317	BoLA-DRB3_014_01_02	GGSCVAIAPAFQEYA	VAIAPAFQE	1.72	42443
317	BoLA-DRB3_020_01_01	GGSCVAIAPAFQEYA	VAIAPAFQE	1.84	42443
317	BoLA-DRB3_020_01_02	GGSCVAIAPAFQEYA	VAIAPAFQE	1.84	42443
317	BoLA-DRB3_020_09	GGSCVAIAPAFQEYA	VAIAPAFQE	1.84	42443
317	BoLA-DRB3_020_10	GGSCVAIAPAFQEYA	VAIAPAFQE	1.84	42443
317	BoLA-DRB3_033_01	GGSCVAIAPAFQEYA	VAIAPAFQE	1.86	42443
317	BoLA-DRB3_007_02	GGSCVAIAPAFQEYA	VAIAPAFQE	1.87	42443
317	BoLA-DRB3_077_01	GGSCVAIAPAFQEYA	VAIAPAFQE	1.98	42443

Peptides having ≤ 2% rank in a frame of 15 aa for a panel of 135 BoLA-DR molecules.
